# Retrospective Study of the Clinical Profile and Prognostic Indicators in Patients of Alcoholic Liver Disease Admitted to a Tertiary Care Teaching Hospital in Western Nepal

**DOI:** 10.4103/1319-3767.54746

**Published:** 2009-07

**Authors:** Om K. Pathak, Raju Paudel, Om B. Panta, Hom P. Pant, Bishnu R. Giri, Baikuntha Adhikari

**Affiliations:** Department of Medicine, Manipal Teaching Hospital, Nepal

**Keywords:** Alcoholic liver disease, demographic profile, prognostic indicators, Nepal

## Abstract

**Background/Aim:**

Alcohol is the most common substance abused in Nepal. Liver disease caused by alcohol abuse, including its end stage, cirrhosis, is a major health care problem, which is difficult to treat.

**Objectives:**

To study the demographic profile, laboratory parameters, complications and their prognostic implications among patients of alcoholic liver disease (ALD).

**Materials and Methods:**

Records of all patients of ALD admitted from January 1' 2005 to December 31' 2006 were studied and followed up to December 31, 2007. A total of 181 patients were analyzed. Their clinical profile and laboratory parameters were noted and analyzed using SPSS-10.0 software.

**Results:**

Among the 181 patients, 80.7% were male, 30.9% were army/ex-army and 65.2% were documented smokers. The mean age of presentation was 52.08 years. Jaundice (57.5%) was the most common presentation followed by hepatomegaly (51.4%). Hypoalbuminemia (50.3) followed by ascites (48.1) were common complications. Death occurred in 19.1% of the patients, the most common cause being hepatic encephalopathy (72.2%) followed by variceal bleeding and hepatorenal syndrome. Jaundice, ascites and hepatic encephalopathy at presentation and female sex were significantly associated with increased mortality along with discriminant score >32, aspartate aminotransferase (AST): Alanine aminotranferase (ALT) ≥ 2, ultrasonography (USG)-proven cirrhosis, rise in prothrombin time ≥5 s, total bilirubin ≥ 4mg/dL and ESR ≥34.

**Conclusion:**

ALD was predominantly seen among the productive age group with a high morbidity and mortality. Jaundice, ascites, hepatic encephalopathy at presentation and female sex are poor prognostic indicators along with discriminant score > 32, AST:ALT ≥ 2, USG-proven cirrhosis, coagulopathy, hyperbilirubenemia and high ESR.

Alcohol is the most common substance abused in Nepal. In a study carried out in 2000 AD, it was found that about 60% of the Nepalese population had experienced alcohol and 41% had taken it during the last 12 months.[1] Alcohol is consumed at some time in their life by 80% of the population.[2] Alcohol is associated with high morbidity and mortality: 3.7% of the global deaths and 4.4% of the global DALYs lost in the year 2002 could be attributed to this exposure.[3] Chronic and excessive alcohol ingestion is one of the major causes of liver disease. Alcoholic liver disease (ALD) is the most common cause of cirrhosis in the Western world.[4]

Although some of the physical findings, especially of alcohol abuse and feminization, are more commonly observed in ALD patients than in other liver diseases, no single physical finding or constellation of findings is 100% specific or sensitive for ALD.[5] Moreover, both laboratory abnormalities and physical findings may be minimal or absent even in patients with established ALD.[6]

Although liver biopsy is the gold standard in the diagnosis of ALD,[6] it is rarely performed in clinical practice. This is because a combination of clinical and laboratory data can make an accurate diagnosis of ALD with the prebiopsy diagnosis of ALD being 98% specific and 79% sensitive.[7,8] Moreover, coagulopathy in patients of ALD makes liver biopsy difficult and risky.

## Objectives

The objectives of the study were to study the demographic profile, laboratory parameters, complications and their prognostic implications among patients of ALD.

## MATERIALS AND METHODS

This retrospective hospital record-based study was conducted in the Manipal teaching hospital, a tertiary care teaching hospital in the western region of Nepal. All ALD patients admitted to the hospital from January 1' 2005 to December 31, 2006 were studied and followed up to December 31, 2007. A total of 181 patients were included in the study. A pilot study was carried out with 5% of the patients and a proforma was designed. Case details with patients' particulars, occupation, risk factors, presenting features, laboratory values, complications, comorbidities and cause of death were entered in the predesigned proforma. Pilot samples were also included in the analysis.

As per the recommendations of the American College of Gastroenterology, a case of ALD is diagnosed in patients with a history of significant alcohol intake, physical signs of liver disease and supportive laboratory data.[6] Definitions of other parameters like HE, spontaneous bacterial peritonitis, hepatorenal syndrome, etc. were taken from current medical diagnosis and treatment. Sonographic evidence of hepatomegaly and splenomegaly is based on the report of the radiologists.

In those cases of ALD which were admitted during the study period but had already visited the hospital previously for the same disease, the clinical parameters and laboratory values of their first visit or of the earliest visit whenever the parameters were documented were taken. Complications, which were diagnosed clinically with supportive laboratory evidences, were noted till the end of the follow-up period. A case of ALD who had visited the hospital for an entirely different problem was excluded from the study. Ethical clearance was taken from the ethical clearance board of the institution. Categorical data were analyzed using SPSS-10.0 with binary logistic regression analysis and Kai square test with a confidence interval of 95%. Continuous data were presented in the form of mean and median.

## RESULTS

A total of 181 patients were analyzed, of which 80.7% (146) were male and 19.3% (35) were females. The mean age of presentation in years is 52.08 ± 13.11 and median age is 52 years (IQ: 42–61). Of all the ALD cases, 65.2% (118) were smokers or had smoked at some time in their lifetime. The mean number of admissions per patient during the study period is 1.75 ± 1.26. The mean duration of stay was 13.41 ± 12.57 days. Most of the patients were army/ex-army 30.9% (56), followed by farmers (6.1%) and service (5%), whereas no occupation was documented in 57.5% of the patients.

Jaundice was the most common presentation, which was present in 57.5% (104) of the patients, followed by hepatomegaly (48.6%), ascites (45.3%) and edema (36.5%) [Table 1].

**Table 1:** Clinical presentation

Presentation	No. of patients	Percentage
Jaundice	104	57.5
Hepatomegaly	88	48.6
Ascites	82	45.3
Edema	66	36.5
Malena	47	26
Anorexia	38	21
Hematemesis	31	17.1
Fever	29	16
Hepatic encephalopathy	22	12.2
Abdominal pain	21	11.6
Pallor	19	10.5
Infection	17	9.4
Spider	16	8.8
Splenomegaly	12	6.6
Other bleeding manifestation	11	6.1
Breathlessness	9	5
Sialosis	6	3.3
Caput	4	2.2

The mean hemoglobin, platelet counts (× 10^3^/*μ*L), MCV and ESR were 11.85 ± 3.45 g/dL, 162.49 ± 89.23, 96.42 ± 9.09 fl and 43.12 ± 39.18 mm in the first hour, respectively. Anemia (hemoglobin < 11 g/dL), MCV > 100 fl and platelet count < 150,000/mm^3^ were present in 42.1%, 33.6% and 51.9% patients, respectively. The mean total leukocyte count was 9303.89 ± 4718.38 cells/*μ*L and the mean differential counts of neutrophils and lymphocytes were 78.64 ± 12.22 and 19.77 ± 11.8, respectively. The mean aspartate aminotransferase (AST), alanine aminotranferase (ALT), AST:ALT, total bilirubin, albumin, A:G ratio and prothrombin time (PT) difference were 142.95 ± 158.85 U/L, 81.56 ± 133.7 U/L, 2.27 ± 1.33, 4.05 ± 4.5 mg/dL, 3.22 ± 0.86 g/dL, 0.97 ± 0.51 and 6.29 ± 6.16 s, respectively [Table 2]. An enzyme-linked immunosorbent test for hepatitis B and C viruses was performed in 40 patients, each of which was positive in four patients. The mean Na^+^, K^+^, urea and creatinine were 132.08 ± 8.43 meq/dL, 4.19 ± 1.04 meq/dL, 66.04 ± 64.86 mg/dL and 1.69 ± 1.2 mg/dL, respectively. Hyponatremia (<130 meq/dL), hypokalemia (<3.5 meq/dL), raised urea (>40 mg/dL) and creatinine (>1.5 mg/dL) were present in 33.1%, 27.4%, 49.1% and 39.4% of the patients, respectively.

**Table 2:** Liver function test

Parameters	Mean	SD	Minimum	Maximum	Median	IQ range
AST (U/L)	142.95	158.85	10	1360	99	58-164
ALT (U/L)	81.56	133.7	6	1410	50	30.5-82.5
AST:ALT ratio	2.27	1.33	0.3	7.9	2	1.4-2.69
PT difference (s)	6.29	6.16	0	46.61	4.9	2.22-8.02
Total bilirubin (mg/dL)	4.05	4.5	0.6	27.4	2.2	1.2-5.05
Direct bilirubin	2.53	3.56	0.2	21	1.2	0.4-3
Indirect bilirubin	1.47	1.44	0.1	8.8	0.9	0.6-1.8
Albumin (g/dL)	3.22	0.86	1.4	6.2	3.1	2.6-3.9
A:G ratio	0.97	0.51	0.2	3.1	0.9	0.7-1.2

Of the 124 patients on whom abdominal sonography was performed, 58.9% (73) showed fatty change, 37.1% (46) had cirrhosis, 4% (five) did not show any change while none of the patients had a sonographic evidence of hepatitis. Splenomegaly and ascites were present in 29.8% and 56.8%, respectively. Upper gastrointestinal endoscopy was performed in 88 patients. Grade I, grade II and grade III varices were present in 33% (29), 17% (15) and 8% (7) of the patients, respectively, whereas 42% (37) of the patients had no varices. Associated endoscopic findings of oesophagogastroduodenitis were present in 31.8% (28) patients and gastric and duodenal ulcers were present in 5.7% each.

**Figure 1:**
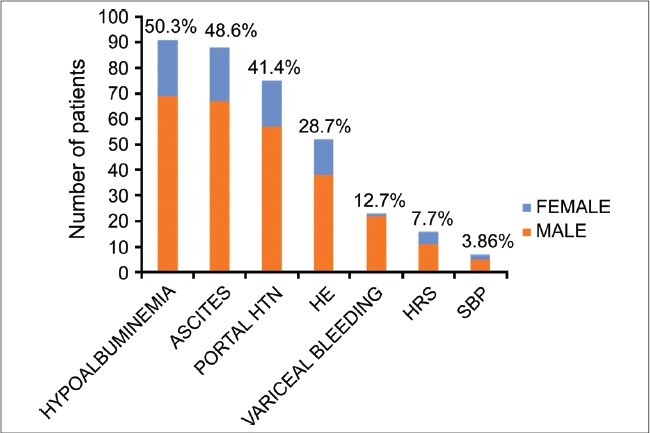
Complications of alcoholic liver disease

Hypoalbuminemia (serum albumin < 3.5 g/dL) was the most common complication (50.3%), which was closely followed by ascites (48.6%) and portal hypertension (41.4%) [Figure 1]. Common comorbidities associated with ALD were esophagogastroduodenitis, hypertension and cholelithiasis, which were present in 17.7%, 17.1% and 14.9% of the patients, respectively.

Of the 181 patients, 36 (19.9%) patients died during the study period and follow-up. Of the 36 patient mortalities, 22 were male (15.1% of male patients) and 14 were females (40% of female patients). Hepatic encephalopathy was the most common cause of death, accounting for 72.2% of the deaths, followed by variceal bleeding (33.3%) and hepatorenal syndrome (33.3%). Sepsis, spontaneous bacterial peritonitis and aspiration pneumonia were less common causes of mortality.

The variables that were significantly associated with mortality in binary logistic regression analysis are presented in Tables 3 and 4. Among clinical presentations, a significant association was established for ascites (*P* < 0.001), jaundice (*P* = 0.002) and hepatic encephalopathy (*P* = 0.003) with mortality during the study and follow-up period. Female sex itself was found to be a poor prognostic indicator, with a *P*-value of 0.001. Among laboratory and sonographic parameters, variables significantly associated with mortality are PT difference between patient and control of ≥5 s (*P* < 0.001), AST: ALT ≥2:1 (*P* = 0.003), total bilirubin ≥4 mg/dL (*P* = 0.004), discriminant function score >32 (*P* < 0.001), ESR ≥34 (*P* = 0.005) and sonographic evidence of cirrhosis (*P* = 0.001) during the study and follow-up period.

**Table 3:** Clinical prognostic indicators

Clinical indicator (at presentation)	OR	95% CI	Significance
Ascites	4.21	2.15–11.21	X^2^ = 15.99, df = 1, *P* < 0.001
Jaundice	3.86	1.59–9.39	X^2^ = 9.8, df = 1, *P* = 0.002
Sex (female)	3.8	1.67–8.48	X^2^ = 11.01, df = 1, *P* = 0.001
HE	2.68	1.07–6.71	X^2^ = 4.72, df = 1, *P* = 0.03

**Table 4:** Laboratory and sonographic prognostic indicators

Lab indicators	OR	95% CI	Significance
PT difference ≥ 5 s	6.63	2.97–14.78	X^2^ = 24.71, df = 1, *P* < 0.001
ESR ≥ 34	3.43	1.4–8.4	X^2^ = 7.84, df = 1, *P* = 0.005
Cirrhosis (USG evidence)	3.58	1.65–7.73	X^2^ = 11.27, df = 1, *P* = 0.001
AST:ALT ≥ 2:1	3.28	1.46–7.33	X^2^ = 8.9, df = 1, *P* = 0.003
Total bilirubin ≥ 4	2.91	1.37–6.2	X^2^ = 8.09, df = 1, *P* = 0.004
DS > 32	4.59	1.95–10.82	X^2^ = 13.17, df = 1, *P* < 0.001

## DISCUSSION

We analyzed 181 patients of ALD, of which the majority (80.7%) were males. A previous study in eastern Nepal showed the male population to be only 59.25%.[9] This may be associated with a variation in the drinking pattern or medical care seeking practice between sexes in these two geographic locations. Our study showed that female sex is significantly associated with increased mortality with a *P*-value of 0.001. This could be due to the gender-dependent differences in the gastric and hepatic metabolism of alcohol added by hormonal factors[2] along with the delayed health care seeking practice among female patients. The mean age of presentation of 52 years shows a high prevalence of the disease among the productive age group, a result similar to previous studies.[9,10] The mean number of hospital admission of 1.75 and the mean duration of hospital stay of 13.41 days indicate a significant burden of the disease. The average patient with ALD loses 12 years of productive life, a much larger loss than that for heart disease (2 years) and cancer (4 years).[11] The risk factors for ALD are duration and quantity of alcohol drinking, hepatitis C virus (HCV) status and nutrition (both malnutrition and obesity).[2,10] However, they were poorly analyzed during clinical practice as shown in our study. This should be looked at with great concern to further strengthen our understanding of factors accelerating ALD. The practice of consuming locally available/home-made alcohol of various concentrations in this part of the world has made it difficult to calculate the amount of alcohol consumed. The high percentage of ALD patients having an occupation of army/ex-army was probably due to the location of the study, Manipal teaching hospital, which covers an area where military services is one of the primary occupations and there is a highly prevalent habit of drinking alcohol among the soldiers.

Jaundice was the most common presenting feature in our study consistent with a previous study carried out in central Nepal.[9] Upper gastrointestinal bleeding and hepatic encephalopathy were less common presentations as compared with a previous study carried out in central Nepal. This may be because we included all patients of ALD in our study whereas the study in central Nepal included only patients of cirrhotic ascites.[9] Probably for the same reason, the mean hemoglobin level was higher, 11.85 g/dL, in our study. In a study carried out in Norway among alcoholic liver cirrhosis, ascites was more common at admission, which was present in 67% of the patients as compared with 45.3% patients in our study.[12] Clinical features like spider angioma, dupuytren's contracture, testicular atrophy, parotid enlargement, gynecomastia, palmer erythema and finger clubbing were uncommon.

In our study, we have found that ALD patients have a high mean ESR value. High ESR ≥34 in the first hour was significantly associated with increased mortality with a *P*-value of 0.005. The exact cause for this association was not known but it may be due to the systemic inflammatory reaction, including hepatitis associated with alcohol consumption along with a higher prevalence of anemia among ALD patients. Various studies showed the increased prevalence of HCV infection in patients of ALD and that HCV infection is significantly associated with the development of advanced ALD. In a study carried out in Pakistan, hepatitis B surface antigen (HBsAg) was found in 12% and HCV antibodies were found in 66% of the patients of hepatic encephalopathy.[13] But, in our study, HBsAg and HCV antibody tests were performed in 40 patients only, reflecting financial constrains in patient management. We also have a lower prevalence of hepatitis B and HCV infection among ALD patients with a 10% positive result each.

The absolute value of AST and ALT in ALD patients is usually <300 u/L.[14] The diagnosis of ALD increases as the AST/ALT ratio increases.[15,16] AST/ALT ratio >2 is suggestive of alcoholic hepatitis.[15] A high AST/ALT ratio also suggests advanced ALD.[10] Our study showed that AST/ALT ≥ 2:1 at presentation is significantly associated with increased mortality, with a *P*-value of 0.003. Coagulopathy is common in patients of ALD. Prolonged PT is associated with increased mortality.[17] Our study showed that the mean rise in PT was 6.29 s while a previous study in Pakistan among patients of hepatic encephalopathy had a PT > 5 s in 44% of the patients.[13] Our study also showed that PT differences ≥5 s was significantly associated with increased mortality with a *P*-value <0.001. Hyperbilirubenemia was more frequently associated with deaths.[17] Our study also showed that total bilirubin >4 mg/dL at presentation was significantly associated with increased mortality, with a *P*-value of 0.004.

Patients with discriminant function >32 have poor prognosis and increased mortality risk, with a *P*-value <0.001, which was consistent with previous studies.[18,19] Cirrhosis was a poor prognostic indicator in patients of ALD.[20] In our study, sonography showed cirrhosis in 37.1% of the patients and ultrasonography-proven cirrhosis has significantly increased the mortality risk, with a *P*-value of 0.001.

Hypoalbuminemia was the most common complication, which was present in 50.3% of the patients of ALD. Hypoalbuminemia was present in 86% of the patients of hepatic encephalopathy in a study carried out in Pakistan.[13] The mean albumin was 3.2 g/dL and the mean A/G ratio was 0.97. Previous study carried out in central Nepal showed that reversal of the A/G ratio can help in the diagnosis of ALD.[16]

Subtle signs of hepatic encephalopathy are observed in nearly 70% of the patients with cirrhosis. Approximately 30% of the patients dying of end-stage liver disease experience significant encephalopathy, approaching coma.[21] Although the exact cause of death in hepatic encephalopathy is not known, it has been reported as the most common cause of death in our study, which accounts for 72.2% of the deaths.

Our study had many limitations. The study was retrospective, so some of the information was missing from the records. Quantitative measurement of alcohol intake was not possible in history because of the wide variety of alcohol in use, including home-made alcoholic beverages that vary greatly in concentration. Although most of the investigations were of first presentations to the hospital, some of them not performed at presentation were taken as soon as they were performed. This hospital record-based study was not able to include the visit of patients to other hospitals for the same disease. Complications and death of patients that occurred outside the study hospital were also not included. The results could have been improved by carrying out a prospective study. The significant association of clinical and laboratory poor prognostic indicators were only binomial and so a multinomial analysis will be required for the generalization of the results of the study.

## CONCLUSION

The result of this study establishes most of the known facts about ALD in the population of our part of world, reinforcing the clinical and laboratory prognostic indicators of ALD. ALD can have a constellation of clinical presentation and none of them is sensitive and specific for detection of ALD. Not only liver function tests, patients of ALD also have abnormal renal function tests and hematological abnormalities. Mostly affecting the productive age group of the male population, ALD has a high economic burden to the society as well. We recommend screening for alcohol abuse in all adult patients presenting to the hospital as early detection of ALD can decrease both morbidity and mortality due to ALD.

## References

[1] Rupa Dhital Alcohol and young people in Nepal.

[2] Dennis Kasper L, Eugene Braunwald, Anthony Fauci S, Stephen Hauser L, Dan Longo L, Larry Jameson J Harrison's Principles Of Internal Medicine.

[3] WHO Sixtieth world health assembly, Provisional agenda item 12.7. April 5 2007. Evidence-based strategies and interventions to reduce alcohol-related harm.

[4] Rehn N, Room R, Edwards G (2001). Alcohol in the European Region – consumption, harm and policies.

[5] Morgan MY, McIntyre N, Benhamou JP, Bircher J (1991). Alcoholic liver disease Natural history, diagnosis, clinical features, evaluation, management, prognosis and prevention. Oxford textbook of clinical Hepatology.

[6] McCullough AJ, O'Connor JF (1998). Alcoholic liver disease: Proposed recommendations for the American College of Gastroenterology. AM J Gastroenterol.

[7] Ryback RS, Eckardt MJ, Felsher B, Rawlings RR, Sanchez GC, Baunsgaard P, Lundborg CJ (1982). Biochemical and hematologicalcorrelates of alcoholism and liver disease. JAMA.

[8] Talley NJ, Roth A, Woods J, Hench V (1988). Diagnostic value of liver biopsy in alcoholic liver disease. J Clin Gastroenterol.

[9] Syed VA, Ansari JA, Karki P, Regmi M, Khanal B Spontaneous bacterial peritonitis in cirrhotic ascitis: A prospective study in a tertiary care hospital, Nepal.

[10] Zhe Shen, You-Ming Li, Chao-Hui Yu, Yi Shen, Lei Xu, Cheng-Fu Xu, Jin-Jin Chen, Hua Ye, Gen-Yun Xu Risk factors for alcohol-related liver injury in the island population of China: A population-based case-control study.

[11] Friedman SL, McQuaid KR, Grendell JH Current Diagnosis and Treatment in Gastroenterology.

[12] Bell H, Jahnsen J, Kittang E, Raknerud N, Sandvik L (2004). Long-term prognosis of patients with alcoholic liver cirrhosis: A 15-year follow-up study of 100 Norwegian patients admitted to one unit. Scand J Gastroenterol.

[13] Saad Maqsood, Amer Saleem, Adeel Iqbal, Javed Aslam Butt (2006). Precipitating factors of hepatic encephalopathy: Experience at Pakistan Institute of Medical Sciences Islamabad. J Ayub Med Coll Abbottabad.

[14] Fairbanks KD Alcoholic liver disease.

[15] Cohen JA, Kaplan MM (1979). The SGOT/SGPT ratio—an indicator of alcoholic liver disease. Dig Dis Sci.

[16] Majhi S, Baral N, Lamsal M, Mehta KD (2006). De Ritis ratio as diagnostic marker of alcoholic liver disease. Nepal Med Coll J.

[17] Maddrey WC, Boitnott JK, Bedine MS, Weber FL, Mezey E, White RI (1978). Corticosteroid therapy of alcoholic hepatitis. Gastroenterology.

[18] Imperiale TF, McCullough AJ (1990). Do corticosteroids reduce mortality from alcoholic hepatitis?. A meta-analysis of the randomized trials. Ann Intern Med.

[19] Carithers RL, Herlong HF, Diehl AM, Shaw EW, Combes B, Fallon HJ (1989). Methylprednisolone therapy in patients with severe alcoholic hepatitis. A randomized, multicenter trial. Ann Intern Med.

[20] Gluud C, Henriksen JH, Nielsen G (1988). Prognostic indicators in alcoholic cirrhotic men. Hepatology.

[21] Ferenci P, Haubrich WS, Schaffner F, Berk JE (1995). Hepatic encephalopathy. Bockus Gastroenterology.

